# A Highlight-Generation Method for Rendering Translucent Objects

**DOI:** 10.3390/s19040860

**Published:** 2019-02-19

**Authors:** Hui Yu, Peter X. Liu, Lingyan Hu

**Affiliations:** 1 School of Information Engineering, Nanchang University, Nanchang 330031, China; steve_hugh@email.ncu.edu.cn; 2 Department of Systems and Computer Engineering, Carleton University, Ottawa, ON K1S 5B6, Canada

**Keywords:** 3D scanning, translucent objects, specular highlight, surface detail preservation, illumination, translucency perception

## Abstract

The acquisition of translucent objects has become a very common task thanks to the progress of 3D scanning technology. Since the characteristic soft appearance of translucent objects is due to subsurface scattering, the details are naturally left out in this appearance. For objects that have complex shapes, this lack of detail is obviously more prominent. In this paper, we propose a method to preserve the details of surface geometry by adding highlight effects. In generating highlight effects, our method employs a local orthonormal frame and combines, in a novel way, the incoming and outgoing light in approximating the subsurface scattering process. When the incident illuminant direction changes from nearly overhead to nearly horizontal, our method effectively preserves complex surface geometry details in the appearance of translucent materials. Through experiments, we show that our method can store surface features as well as maintain the translucency of the original materials and even enhance the perception of translucency. By numerically comparing the generated highlight effects with those generated by the traditional Bidirectional Reflectance Distribution Function (BRDF) models with different bandwidth parameters, we demonstrate the validity of our proposed method.

## 1. Introduction

Advances in 3D scanning technology have been widely applied to object acquisition due to the abundance of low-cost RGB-D sensors. Translucent objects are very common in realistic domestic environments, and by placing a scanner at strategically selected positions, geometric details can be captured with thoroughness and high fidelity. However, in rendering the complex scanned data of objects made from translucent materials, blurred geometric details become more pronounced as the illuminant direction approaches a horizontal orientation. According to previous works [1,2], highlights can contribute to the visual impression of translucency and observers can use the shape information of highlights alone to estimate curvature magnitude. In 3D color printing, a high level of detail and realism should be exhibited for the purpose of reproducing complex appearance properties and more control over the internal structure [3]. To preserve subtle geometry details in the appearance of translucent materials, we developed a method that combines subsurface scattering approximation with generated highlight effects. We applied the directional dipole model with slant illuminant angles to the highlight-generation method and successfully maintained complicated geometry details in the rendering results for translucent materials.

Currently, many specular highlight component expressions of the BRDF (Bidirectional Reflectance Distribution Function) model employ a local orthonormal frame to calculate the highlight value. The tangential vectors of the local orthonormal frame are important for the shape of the generated highlight. Many methods used tangent vectors to simulate highlight and produce special visual effects.

Various approaches [4,5,6] used the frame [x,y,z] to present the directions of the anisotropic highlight, and they showed that surface positions contributing to specular highlights are in planes orthogonal to the half-way vector.

The visual appearance of specified materials and the dimensions of the physical structure are related to the chosen local orthonormal frame. 

Brandt and Scandolo [7] used spectral methods to represent and process tangential vector fields on surfaces, allowing for real-time fur editing on surface meshes with global smoothly varying hair direction. Zinke and Weber [8] used a fiber’s local coordinate system to parameterize light scattering in a filament. They reproduced view-dependent translucency and global highlights in hair fibers. Irawan and Marschner [9] described the appearance of woven cloth that is parameterized by the local orthonormal frame [u,v,r]. The curvature of the yarn segment is controlled by the spine’s tangent vector, and the circular cross-section for each u is featured by v and r. Ament and Dachsbacher [10] made an orthonormal basis at each shading point and in the tangent plane. The generated specular highlight visually emphasized the overall salient structure of the ellipsoids.

These models can describe reflectance on a surface or scattering inside a medium by analyzing specular reflection on specific structure models such as a cylinder, a curved cylinder, or voxels stored in a lattice. However, they are not well suited to surfaces that have complex geometry details. 

Methods to add highlight effects on translucent materials have seldom been reported in the literature due to light scattering away inside a medium. Fleming and Bülthoff [11] used full-scene illumination from Debevec’s light-probe database [12] to add highlights on translucent materials. Motoyoshi [13] also employed the lighting environment used for Debevec’s light-probe high dynamic range images [12] to add natural highlights on translucent materials. Wang et al. [14] added surface shading to heterogeneous translucent materials by using the Cook-Torrance model. In these methods, highlights and subsurface scattering approximation are carried out in two distinct light transport processes and thus do not affect each other. 

Some computer vision methods focus on extracting specular components from captured images. The main idea is to separate the global illumination effect and the directional reflection component to obtain the meso-structure of translucent objects and detailed surface normal vectors.

Chen et al. [15] combined polarization-difference imaging with phase shifting to separate all traces of subsurface structures and recover the geometry of translucent objects. They improved upon this by using modulated phase shifting to separate direct lighting and global light transport [16]. They could more efficiently filter out subsurface scattering and inter-reflections that are still contained in traditional phase shifting.

The drawback of these methods is that they are essentially BRDF-based highlight-generation methods, in addition, they cannot completely separate the subsurface scattering component and the direct-illumination component, and the residual direct component may be too low and noisy compared with the translucent surfaces.

Kim and Ghosh [17] successfully exploited polarized light-field imaging solutions to separate diffusion and specular reflection of human skin, but their method can only process highlights located in inner region of the scattering image.

The method of Sun et al. [18] is based on color information completely without geometrical details of the surface, their method does not depend on polarization, and can remove strong specular highlight, but is tailored for overall smooth regions.

Photometric stereo can also be used to recover surface details for translucent materials [19,20]. However, these algorithms are operated on near flat regions, and so they are not suited to the sharp geometry features of translucent objects. 

Our method simulates the highlight effects on the approximation of subsurface scattering in rendering translucent materials. It can generate highlight effects in the appropriate position for normally shaped surfaces. Moreover, the subtle geometry details are preserved while the translucency is well maintained in the rendering results, and the method can also improve the translucency effects in edge regions.

## 2. Background Theory

The approach of rendering translucent materials using subsurface scattering is based on the radiative transfer equation. Several BSSRDF (Bidirectional Scattering Surface Reflectance Distribution Function) models are available to estimate subsurface scattering, including the standard dipole model [21], the quantized diffusion model [22], the better dipole model [23], and the directional dipole model [24]. We chose the directional dipole model for rendering translucent materials because it considers the directions of incoming and outgoing light of the light-scattering process in the estimation of BSSRDF, and it also captures translucency effects that are present in the full path tracing and geometry details by directional illuminant light, both of which remain absent in other models. 

For generating highlight effects, we employ the specular component expression of the Ward [25], Ashikhmin [26], and Lafortune [27] models because they all apply a local orthonormal frame and bandwidth parameters to control the shape of highlights. When computing highlights, the NDF (normal distribution function) is the main source in simulating special highlight effects in microfacet models. By filtering NDF, Kaplanyan [28] found small highlights across an entire pixel footprint. Guo et al. [29] introduced the extended NDF to express visually perceived roughness due to reflections and refractions. In microfacet theory, M is a function combining Fresnel, shadowing and masking term, which represents the projected area that is visible from both the incoming and outgoing direction. Progress has been made on modeling multiple specular reflections in the v-groove cavity. Lee et al. [30] defined the shadow masking term to show single scattering and multiple scattering inside the v-groove, their result illustrates a more specular appearance due to multiple scattering inside v-groove for high roughness values. Xie and Hanrahan [31] derived the formula for M and NDF, with the generated highlight, their BRDF eliminated the dimmer appearance of rough surfaces and took on a more saturated color in the rendering images. The shape and intensity information of highlights are highly related to the directions of the local orthonormal frame. For objects placed in special positions, for example, the hairs located along the surface tangent vector [4], the surface positions that can contribute to specular highlight lie in planes orthogonal to the half-way vector of the incoming and outgoing light direction; these are highlights that occur when the half-way vector is perpendicular to the surface tangent vector. When more complex shapes, such as a cylinder or a curved cylinder, are used to model an object’s shape, the local orthonormal frame describes the scattering geometry of light entering or exiting the medium, and the highlight position is distributed on the elevation and azimuthal angles of the illumination directions.

## 3. Highlight Effects Generation Methods

Our method is inspired by the concept of using a minimum enclosing cylinder to model a fiber for approximating outgoing radiance at a surface patch from incoming irradiance at another patch by scattering effects [8]. We employed a directional dipole model to render the translucent appearance due to subsurface scattering of light from the incident point to the exitant point. Our goal is thus to define the highlight effects using the incident and emergent light at two different points in the approximation of subsurface scattering. By the addition of the generated highlight effects, the blurry structural details of translucent objects should become clear in the rendering result.

**Figure 1:**
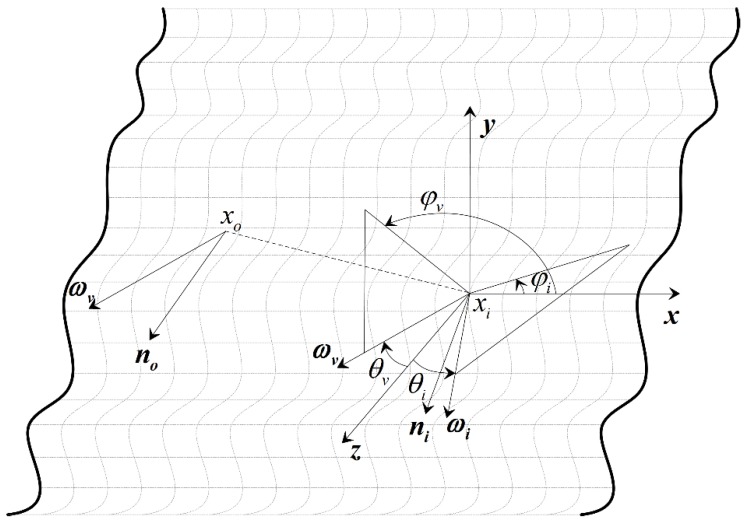
Geometry of light transport between incident and exitant points on the surface and the model used to calculate highlight effects.

The geometry of light transport is shown in Figure 1. Here, an incident light from direction $-\omega_{i}$ enters the material at surface point $x_{i}$, gets refracted and scattered inside the medium, and goes out at exitant point $x_{o}$. 

We define the highlight effect that one would observe from $\omega_{o}$ at exitant point $x_{o}$ in the subsurface scattering process, in which the directional light is incident on $x_{i}$ from $\omega_{i}$. The idea behind this is that BSSRDF and BRDF both describe the observed radiance contributed by the incident irradiance. In BSSRDF, the contribution is by subsurface scattering, while in BRDF it is by reflection. We use the specular highlight expression of three BRDF models to calculate our highlight effects.

Our method consists of four steps: (1) defining the local orthonormal frame, (2) choosing the illumination directions, (3) selecting the highlight component expression, and (4) generating highlight effects by a Monte Carlo method for rendering translucent materials. 

### 3.1. Defining the Local Orthonormal Frame

We define half-way vector h as the normalized vector of the sum of incoming light $\omega_{i}$ at incident point $x_{i}$ and outgoing light $\omega_{o}$ at exitant point $x_{o}$.

(1) $$h=(\omega_{i}+\omega_{o})/\left|\omega_{i}+\omega_{o}\right|$$

We employ the local orthonormal frame [t,b,n] at $x_{i}$ in the highlight-calculation expression. To simplify the calculation, we define [t,b,n] as follows: n is the normal vector at $x_{i}$, x is the x axis of the Cartesian coordinate system, t is the cross-product of n and x, and b is the cross-product of n and t.

The definition of h and the choice of position $x_{i}$ to define [t,b,n] are important in our method. When $x_{i}$ is close to $x_{o}$, we assume the change in viewing vectors from these two points is trivial and use the viewing vector $\omega_{o}$ to calculate the highlight effects at $x_{i}$. 

In the calculation, $n_{t}$ and $n_{b}$ are the bandwidth parameters used to control the projection magnitude in the t and b directions. By using a different combination of bandwidth parameters, we can obtain varying highlight shapes for areas with complex geometry details and, moreover, enhance the visual perception of details in these areas.

### 3.2. Choosing the Illumination Directions

We generate highlight effects from different incident illumination directions. When we use the directional dipole model to approximate the diffusive part of the BSSRDF, the calculation process assumes that the diffusive light at the exitant point no longer depends on outgoing direction $\omega_{o}$. To effectively analyze the complex shape of the generated highlight behavior, we choose elevation and azimuth angles of the incoming illumination and different bandwidth parameters $n_{t}$ and $n_{b}$ to generate highlight effects. We set fixed elevation and azimuth angles for different sampled incident points.

### 3.3. Selecting the Highlight Component Expression

Based on the microfacet model, the specular highlight component $\rho_{s}$ of BRDF or BSDF (Bidirectional Scattering Distribution Function) can be expressed by

(2) $$\rho_{s}(i,o)=M(i,o)D(i,o)$$

where M is a function combining Fresnel and shadowing terms and D is the normal distribution function. 

For visualizing different appearance effects, we employ the specular highlight component of the Ward, Ashikhmin, and Lafortune models to generate the highlight effects. The normalized tangent vectors t and b specify the principal directions of the highlight. The Ashikhmin and Ward models are microfacet models, and they require parameters $n_{t}$, $n_{b}$ to control the projection of h to tangent vectors t and b. Specifically, for the Ward model, we use this expression:

(3) $$M(\omega_{i},\omega_{o})=1/(4\pi \sqrt{\frac{2.0}{n_{t}}}\sqrt{\frac{2.0}{n_{b}}}\sqrt{\left(\omega_{i}.n)(\omega_{o}.n\right)})$$

(4) $$D(h)=e^{-\frac{2}{1+h.n}(\frac{n_{t}{\left(h.t\right)}^{2}}{2.0}+\frac{n_{b}{\left(h.b\right)}^{2}}{2.0})}$$

For the Ashikhmin model, we use the highlight expression with

(5) $$M(\omega_{i},\omega_{o})=\frac{\sqrt{\left(n_{t}+1)(n_{b}+1\right)}F(\omega_{i}.h)}{8\pi (\omega_{i}.h).max(\omega_{i}.n,\omega_{o}.n)}$$

(6) $$D(h)=(h.n)\frac{n_{t}{\left(h.t\right)}^{2}+n_{b}{\left(h.b\right)}^{2}}{1-{\left(h.n\right)}^{2}}$$

where F is a Fresnel term that controls the proportion of reflected light.

For the Lafortune model, we need $n_{t},n_{b},n_{n}$ to control the shape of the highlight, and the expression is

(7) $$f_{s}(\omega_{i},\omega_{o})={\left(n_{t}\omega_{i}{}_{t}.\omega_{o}{}_{t}+n_{b}\omega_{i}{}_{b}.\omega_{o}{}_{b}+n_{n}\omega_{i}{}_{n}.\omega_{o}{}_{n}\right)}^{k}$$

where $\left[\omega_{it},\omega_{ib},\omega_{in}\right]$ is incoming light $\omega_{i}$ defined in the local orthonormal frame [t,b,n], $\left[\omega_{ot},\omega_{ob},\omega_{on}\right]$ is outgoing light $\omega_{o}$ defined in [t,b,n], and k is the exponent parameter that determines the size and shape of the highlights.

### 3.4. Generating Highlight Effects by A Monte Carlo Method

Since the subsurface scattering decreases exponentially with distance r of the incident and exitant points, we multiply an exponential term to constrain the highlight effects within a small-scale distance.

In our rendering of translucent materials, the BSSRDF is implemented in a Monte Carlo ray tracer. We fix $x_{o}$ and sample different incoming points $x_{i}$ to approximate the highlight effects at $x_{i}$. We then estimate the highlight effects at $x_{o}$ by averaging the highlight values of $x_{i}$. In summary, for methods based on the Ward and Ashikhmin models, the highlight effects’ expressions at $x_{o}$ in a Monte Carlo estimator are defined as

(8) $$\frac{1}{N}\sum_{i=1}^{N}\frac{M(\omega_{i},\omega_{o})D(\omega_{i},\omega_{o})e^{-\sigma r}}{pdf(x_{i})}$$

for method based on the Lafortune model, the highlight effects’ expression is

(9) $$\frac{1}{N}\sum_{i=1}^{N}\frac{f_{s}{(\omega }_{i}{,\omega }_{o}{)e}^{-\sigma r}}{{pdf(x}_{i})}$$

where N is the sampling number of incident lights, $pdf(x_{i})$ is the probability of sampling a mesh point on the model surface, and σ is the extinction coefficient of the rendering material. The generated highlight effects by the Monte Carlo estimator should be added to the color value of the final pixel that is estimated by the directional dipole model. The proposed method is reciprocal for the three selected highlight component expressions.

## 4. Experiment Setup

**Figure 2:**
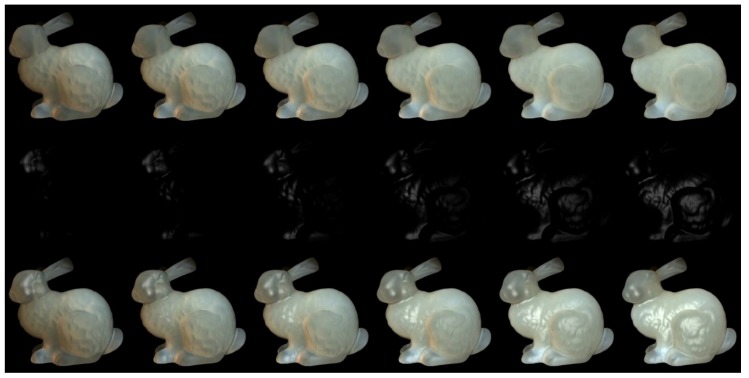
Visual appearance of rendering of chocolate milk (regular) under slant illuminant angles from nearly overhead to nearly horizontal. From left to right, the elevation angles are 10°, 25°, 40°, 55°, 70°, and 85°. First row: rendering results by directional light without adding highlight effects. Second row: generated highlight effects under the same illuminant conditions of the first row. Third row: rendering results by adding highlight effects of the other two rows at the same elevation angles.

The proper choice of the illuminant direction is important for preserving details when adding highlight effects. We set the upper hemisphere with its origin at the surface incident point and its north pole as vector [0,0,1] (Z axis). Therefore, in spherical coordinates, the illuminant direction can be described by elevation angle θ and azimuthal angle φ. To choose the incident illuminant directions of the incident points, we substitute the object surface that is visible to the viewer with the XOY plane in a simplified way. According to Adams and Elder [32], as the illuminant direction changes from directly overhead to horizontal, the shape becomes more ambiguous and the perceived convexity due to the highlight effect becomes pronounced. To verify this phenomenon, we choose six elevation angles of 10°, 25°, 40°, 55°, 70°, and 85°. Because the general ambiguous condition of the details is not evidently affected by the azimuthal angle, we choose an azimuthal angle of 25° in our first experiment (results in Figure 2).

To compare the results of the generated highlight effects by our model and those by traditional models, we used parameters θ=85 ${}^{\circ }$, φ=85 ${}^{\circ }$, which is a nearly horizontal illumination direction, for the incident light direction in the following experiments. The viewpoint is fixed, and the exitant light direction is from the exitant point to the eye. In the computation of specular highlight, we make sure the angle between the incident light and the normal vector at the incident point is less than 90°. For some small incident elevation angles, the angles are more than 90°, so the highlight result is set to zero in these regions.

**Figure 3:**
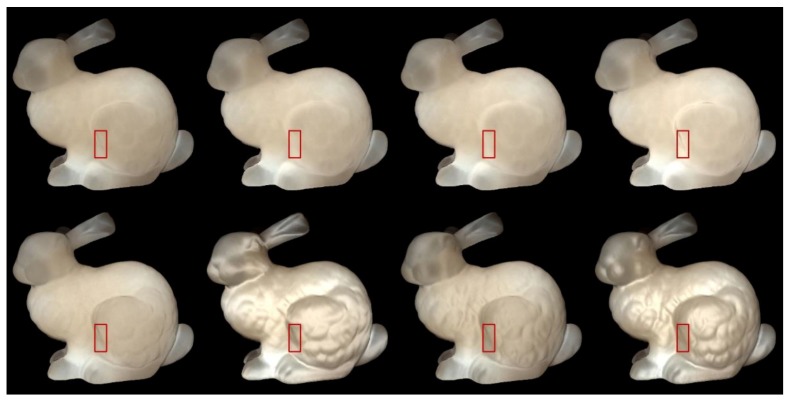
Comparison of highlight effects generated by our Ward-based method (first row) and the method based on the traditional Ward model (second row) for marble material. From left to right, the $n_{t}$, $n_{b}$ parameters are $n_{t}$ is 2, $n_{b}$ is 2; $n_{t}$ is 2, $n_{b}$ is 10; $n_{t}$ is 10, $n_{b}$ is 2; $n_{t}$ is 10, $n_{b}$ is 10.

**Figure 4:**
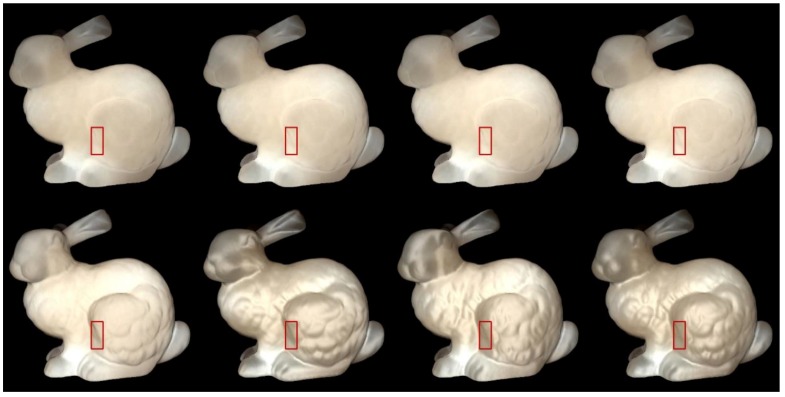
Comparison of highlight effects generated by our Ashikhmin-based method (first row) and the method based on the traditional Ashikhmin model (second row) for marble material. From left to right, the $n_{t}$, $n_{b}$ parameters are $n_{t}$ is 2, $n_{b}$ is 2; $n_{t}$ is 2, $n_{b}$ is 10; $n_{t}$ is 10, $n_{b}$ is 2; $n_{t}$ is 10, $n_{b}$ is 10.

**Figure 5:**
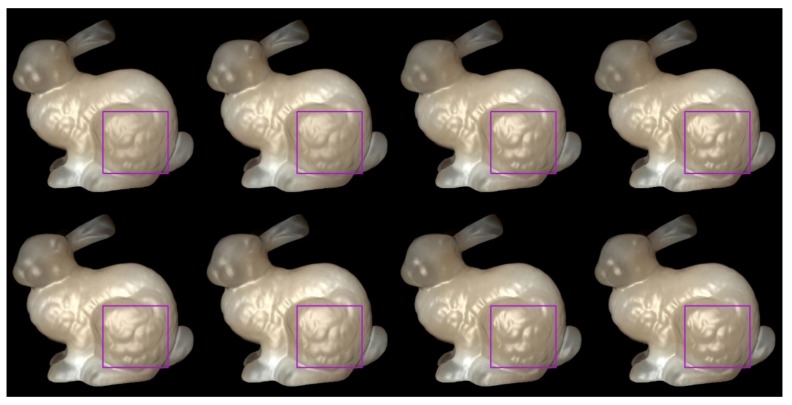
Comparison of highlight effects generated by our Lafortune-based method (first row) and the method based on the traditional Lafortune model (second row) for marble material. From left to right, the $n_{t}$, $n_{b}$ parameters are $n_{t}$ is 0.2, $n_{b}$ is 0.5; $n_{t}$ is 0.5, $n_{b}$ is 0.2; $n_{t}$ is −0.2, $n_{b}$ is −0.5; $n_{t}$ is −0.5, $n_{b}$ is −0.2.

**Figure 6:**
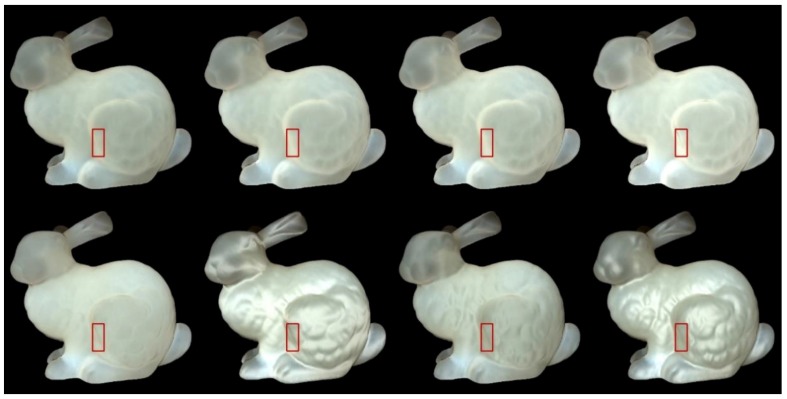
Comparison of highlight effects generated by our Ward-based method (first row) and the method based on the traditional Ward model (second row) for chocolate milk (regular) material. From left to right, the $n_{t}$, $n_{b}$ parameters are $n_{t}$ is 2, $n_{b}$ is 2; $n_{t}$ is 2, $n_{b}$ is 10; $n_{t}$ is 10, $n_{b}$ is 2; $n_{t}$ is 10, $n_{b}$ is 10.

**Figure 7:**
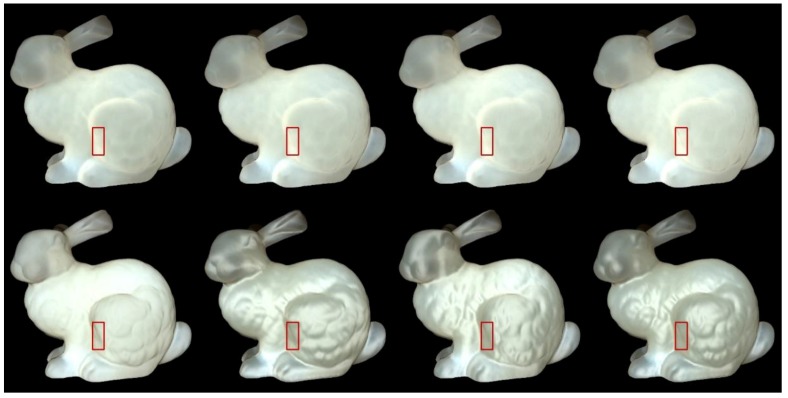
Comparison of highlight effects generated by our Ashikhmin-based method (first row) and the method based on the traditional Ashikhmin model (second row) for chocolate milk (regular) material. From left to right, the $n_{t}$, $n_{b}$ parameters are $n_{t}$ is 2, $n_{b}$ is 2; $n_{t}$ is 2, $n_{b}$ is 10; $n_{t}$ is 10, $n_{b}$ is 2; $n_{t}$ is 10, $n_{b}$ is 10.

**Figure 8:**
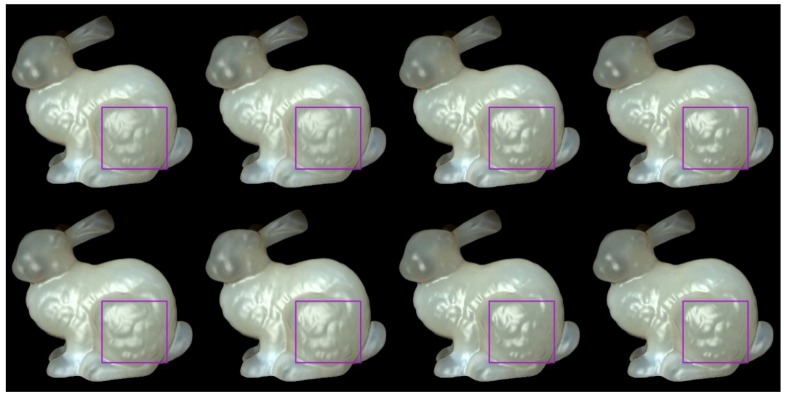
Comparison of highlight effects generated by our Lafortune-based method (first row) and the method based on the traditional Lafortune model (second row) for chocolate milk (regular) material. From left to right, the $n_{t}$, $n_{b}$ parameters are $n_{t}$ is 0.2, $n_{b}$ is 0.5; $n_{t}$ is 0.5, $n_{b}$ is 0.2; $n_{t}$ is −0.2, $n_{b}$ is −0.5; $n_{t}$ is −0.5, $n_{b}$ is −0.2.

We used Stanford bunny object with two materials which are from Jensen et al. [21] and Narasimhan et al. [33] in our experiment: marble and chocolate milk (regular). Our method can preserve the details of both the solid and liquid translucent material. In all experiments, the resolution of the resulting image is 512 × 512 pixels, and the maximum intensity of the generated highlight is set to 0.35. For marble material, in Figure 3 and Figure 4, the selected region (red closeups) has 1440 pixels, in Figure 5, the selected region (purple closeups) has 20,720 pixels. For chocolate milk (regular) material, in Figure 6, Figure 7 and Figure 8, the selected regions are the same as in the marble material. In every row of Figure 3, Figure 4, Figure 6 and Figure 7 from left to right, the $n_{t}$, $n_{b}$ parameters are $n_{t}$ is 2, $n_{b}$ is 2; $n_{t}$ is 2, $n_{b}$ is 10; $n_{t}$ is 10, $n_{b}$ is 2; $n_{t}$ is 10, $n_{b}$ is 10. In the results of Lafortune-based methods (Figure 5; Figure 8), the exponent parameter *k* is 10. $n_{t}$ is 1, and, from left to right, the $n_{t}$, $n_{b}$ parameters are $n_{t}$ is 0.2, $n_{b}$ is 0.5; $n_{t}$ is 0.5, $n_{b}$ is 0.2; $n_{t}$ is −0.2, $n_{b}$ is −0.5; $n_{t}$ is −0.5, $n_{b}$ is −0.2.

## 5. Results

### 5.1. Rendering Results under Slant Illumnation

When the illumination direction changes from nearly overhead to nearly horizontal (Figure 2), the soft characteristic appearance of the chocolate milk (regular) material become more pronounced, so the geometry details become more ambiguous. On the other hand, the shape of the highlights become wider. The blurry structural details become clearer by adding the generated highlight effects, and this result becomes pronounced when the elevation angles approach the horizontal direction. 

### 5.2. Comparision of the Proposed Method to Traditional Methods

In Figure 3; Figure 6, the presented method has an advantage in the perception of edge over the method based on the traditional Ward model. The left edge of the bunny leg is seen more clearly by adding highlight effects in the proposed method. According to [11], the bright appearance around the edge is due to light passes through the thin edges and vertices. The proposed method also preserves the structural details of the bunny’s leg region, although the visual appearance is not clearer than the results produced by the method based on the traditional Ward model under some conditions (fourth columns of Figure 3; Figure 6). However, the proposed method maintains the translucency of the material while the results generated by the method based on the traditional Ward model loses its tr anslucency to some degree and the surface becomes a metal-like appearance, especially in the cases when is 2, is 10.

The results in Figure 4; Figure 7 are similar to those of the method based on the Ward model. The presented method keeps the shape of the left edge of the bunny’s leg and preserves the details of the bunny’s leg region to some degree. However, the condition of the preserved details is not more prominent than the result of the method based on the traditional Ashikhmin model. The side effects of the method based on the traditional Ashikhmin model, for example when $n_{t}$ is 2, $n_{b}$ is 2 on the bunny’s chest region, imply that the highlight effects are too strong and the geometry details are washed out in that region. When $n_{t}$ is 2, $n_{b}$ is 10, and when $n_{t}$ is 10, $n_{b}$ is 2; here, the results display a more metal-like appearance, and the translucency is lost to some degree.

As shown in Figure 5; Figure 8, the proposed method and the compared method based on the traditional Lafortune model both generate highlight effects on complex detailed regions, such as the chest and leg regions, and the geometry details are well preserved in these regions. With the generated highlight effects, the translucent object looks more realistic in visual perception. All these highlight-generation methods have succeeded in preserving the geometry details and maintaining translucency to some degree. Among them, the methods based on the Lafortune model have the best visual appearance in surface details and the materials’ translucency. For methods based on the Lafortune model, the shape of the highlights is wider than those based on the Ward and Ashikhmin models, the translucency is well maintained, and it avoids excessively bright or dimmer appearance in the rendering results.

## 6. Discussion

Qi et al. [34] investigated five image statistics of highlight areas to show how these metrics are related to the perceived gloss. We chose two metrics: (1) number of pixels, which corresponds to "percentage of highlight area" and, notably, is highly correlated with perceived gloss in the work of Qi et al. [34]; (2) average intensity of generated highlight, which is used to analyze the perceived quality of the covered regions. We set the intensity threshold value to 0.20, and in Table 1 and Table 2, Number means the number of pixels that have intensity above the threshold value, while Intensity means the average intensity of highlight pixels that have an intensity value higher than the threshold value.

For methods based on the Ward and Ashikhmin models, we chose the left edge of the bunny leg’s for numerical analysis (red closeups in Figure 3, Figure 4, Figure 6, and Figure 7). From Table 1 and Table 2, the proposed method generated stronger highlight effects in number and average intensity at this edge, and the shape of this edge is well preserved and average intensity at this edge, and the shape of this edge is well preserved and visually enhanced. On the contrary, in the results generated by the methods based on the traditional Ward and Ashikhmin models, the values are smaller and the highlight effects at this edge are not as obvious as in the proposed method.

For chocolate milk (regular) material, the results of the generated highlight show similar effects as those for the marble material. From the “shadow-haze” view described in the work of Fleming and Bülthoff [11], our method generates a better cue for the perception of translucency, that is, the superimposition of a sharpened edge while blurring the combined translucent colors.

For methods based on the Lafortune model, we chose the bunny’s leg region for numerical analysis (purple closeups in Figure 5; Figure 8). From Table 1 and Table 2, in the proposed method, when $n_{t}$ is 0.2, $n_{b}$ is 0.5, and when $n_{t}$ is 0.5, $n_{b}$ is 0.2, and it is evident that the values of number and average intensity are smaller than those in cases when $n_{t}$ is −0.2, $n_{b}$ is −0.5 and when $n_{t}$ is −0.5, $n_{b}$ is −0.2. This can be seen from the weaker appearance intensity of the preserved bunny leg’s details in the rendering results; In the results of methods based on the traditional Lafortune model, when $n_{t}$ is 0.2, $n_{b}$ is 0.5 and when $n_{t}$ is 0.5, $n_{b}$ is 0.2, and the values of number are distinctly larger and the values of average intensity are a little smaller than the values in cases when $n_{t}$ is −0.2, $n_{b}$ is −0.5 and when $n_{t}$ is −0.5, $n_{b}$ is −0.2. 

**Figure 9:**
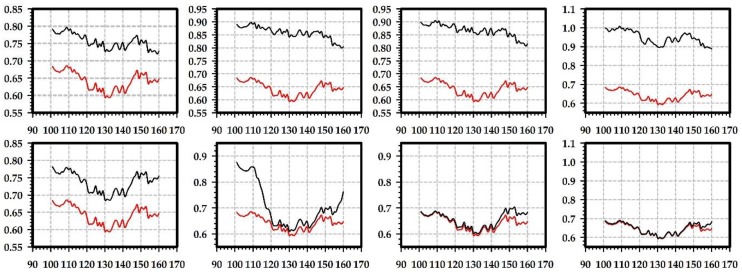
Illustration of generated highlight intensity distribution for the selected column of pixels with the marble material. First row: highlight effects generated by our Ward-based method, where images from left to right correspond to images of the first row in Figure 3; second row: highlight effects generated by the method based on the traditional Ward model, where images from left to right correspond to images of the second row in Figure 3 and the $n_{t}$, $n_{b}$ parameters are $n_{t}$ is 2, $n_{b}$ is 2; $n_{t}$ is 2, $n_{b}$ is 10; $n_{t}$ is 10, $n_{b}$ is 2; $n_{t}$ is 10, $n_{b}$ is 10. In each image, the x axis is the vertical coordinate position of a pixel in the selected line and the y axis is the intensity value of the pixel.

**Figure 10:**
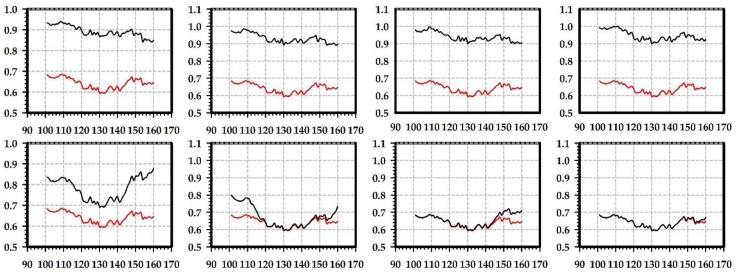
Illustration of generated highlight intensity distribution for the selected column of pixels with the marble material. First row: highlight effects generated by our Ashikhmin-based method, where images from left to right correspond to images of the first row in Figure 4; second row: highlight effects generated by the method based on the traditional Ashikhmin model, where images from left to right correspond to images of the second row in Figure 4 and the $n_{t}$, $n_{b}$ parameters are $n_{t}$ is 2, $n_{b}$ is 2; $n_{t}$ is 2, $n_{b}$ is 10; $n_{t}$ is 10, $n_{b}$ is 2; $n_{t}$ is 10, $n_{b}$ is 10. In each image, the x axis is the vertical coordinate position of a pixel in the selected line and the y axis is the intensity value of the pixel.

We select a vertical line of pixels that is in the middle of the red closeup region (Figure 3, Figure 4, Figure 6 and Figure 7) to study the improvement of translucency by the generated highlight. In Figure 9 and Figure 10, the red line shows approximated subsurface scattering without adding highlights, and the black line shows approximated subsurface scattering with highlights. Our methods and the traditional methods all maintain the peaks of the subsurface scattering values after adding the highlight values. In our methods, the generated highlight has strong values when the value of the subsurface scattering is low, on the other hand, in the traditional methods, the generated highlight has relatively weaker values when the value of the subsurface scattering is low. For the evaluated positions overall, our method has stronger generated highlight values, and the range (difference between maximal and minimal values) of the subsurface scattering values becomes smaller after adding highlight values. 

## 7. Conclusions

We presented a method to preserve surface details by adding highlight effects in the rendering of translucent materials. The method employs the light paths of the incident and exitant points in the light-scattering process, uses the local orthonormal frame in the incident point, combines three highlight-generation models that employ local tangent vectors, generates highlights in appropriate positions, stores surface details under different conditions while maintaining the original material’s translucency, and enhances the translucency in edge regions. All these features help to prove the validity of our method.

We compared the results of the proposed method and traditional models in three highlight expressions. Generally, the proposed method exhibits rich visual effects in regions with complex surface details and geometry features, and it increases the perception of translucency in those regions. Under some conditions, the number and average intensity of the generated highlight effects by the proposed method are smaller than the results generated by the traditional models, i.e., lower values, and the region’s details are less clear than those for higher values. However, the proposed method has the advantage of avoiding excessively bright or metal-like highly specular appearances in the rendering results.

Our method works with a single-direction light, and it can be incorporated in applications that need a fast solution. However, due to the restrictions on incident light directions, in some positions, where the angle between the incident light and the normal vector is above 90°, the highlight value is clamped, so it cannot produce highlight effects in these positions. In the future, we plan to study the relation of the generated highlight position and the local orthonormal frame. Consequently, by applying proper tangent vectors, it would be possible to produce specific highlight effects that are in accordance with a specific geometric shape.

## Figures and Tables

**Table 1:** Number and Intensity of generated highlight for marble material.

Material	Method	Parameters	Numbers	Intensity
marble	Ward-based (proposed)	*n_t_* = 2, *n_b_* = 2	0	0
		*n_t_* = 2, *n_b_* = 10	908	0.2303
		*n_t_* = 10, *n_b_* = 2	987	0.2371
		*n_t_* = 10, *n_b_* = 10	1440	0.2930
	Ward-based (traditional)	*n_t_* = 2, *n_b_* = 2	0	0
		*n_t_* = 2, *n_b_* = 10	4	0.2037
		*n_t_* = 10, *n_b_* = 2	0	0
		*n_t_* = 10, *n_b_* = 10	0	0
marble	Ashikhmin-based (proposed)	*n_t_* = 2, *n_b_* = 2	1326	0.2471
		*n_t_* = 2, *n_b_* = 10	1440	0.2832
		*n_t_* = 10, *n_b_* = 2	1440	0.2903
		*n_t_* = 10, *n_b_* = 10	1440	0.3005
	Ashikhmin-based (traditional)	*n_t_* = 2, *n_b_* = 2	321	0.2257
		*n_t_* = 2, *n_b_* = 10	0	0
		*n_t_* = 10, *n_b_* = 2	0	0
		*n_t_* = 10, *n_b_* = 10	0	0
marble	Lafortune-based (proposed)based	*n_t_* = 0.2, *n_b_* = 0.5	862	0.2176
		*n_t_* = 0.5, *n_b_* = 0.2	1482	0.2266
		*n_t_* = −0.2, *n_b_* = −0.5	2704	0.2577
		*n_t_* = −0.5, *n_b_* = −0.2	2828	0.2601
	Lafortune-based (traditional)	*n_t_* = 0.2, *n_b_* = 0.5	3560	0.2562
		*n_t_* = 0.5, *n_b_* = 0.2	3521	0.2564
		*n_t_* = −0.2, *n_b_* = −0.5	2517	0.2635
		*n_t_* = −0.5, *n_b_* = −0.2	2494	0.2604

**Table 2:** Number and Intensity of generated highlight for chocolate milk (regular) material.

Material	Method	Parameters	Numbers	Intensity
Chocolate milk (regular)	Ward-based (proposed)	*n_t_* = 2, *n_b_* = 2	0	0
		*n_t_* = 2, *n_b_* = 10	482	0.2167
		*n_t_* = 10, *n_b_* = 2	747	0.2254
		*n_t_* = 10, *n_b_* = 10	1440	0.2858
	Ward-based (traditional)	*n_t_* = 2, *n_b_* = 2	0	0
		*n_t_* = 2, *n_b_* = 10	15	0.2038
		*n_t_* = 10, *n_b_* = 2	0	0
		*n_t_* = 10, *n_b_* = 10	0	0
Chocolate milk (regular)	Ashikhmin-based (proposed)	*n_t_* = 2, *n_b_* = 2	1363	0.2514
		*n_t_* = 2, *n_b_* = 10	1440	0.2743
		*n_t_* = 10, *n_b_* = 2	1440	0.2851
		*n_t_* = 10, *n_b_* = 10	1440	0.3078
	Ashikhmin-based (traditional)	*n_t_* = 2, *n_b_* = 2	333	0.2277
		*n_t_* = 2, *n_b_* = 10	0	0
		*n_t_* = 10, *n_b_* = 2	0	0
		*n_t_* = 10, *n_b_* = 10	0	0
Chocolate milk (regular)	Lafortune-based (proposed)	*n_t_* = 0.2, *n_b_* = 0.5	1923	0.2347
		*n_t_* = 0.5, *n_b_* = 0.2	2387	0.2424
		*n_t_* = −0.2, *n_b_* = −0.5	2931	0.2653
		*n_t_* = −0.5, *n_b_* = −0.2	2994	0.2652
	Lafortune-based (traditional)	*n_t_* = 0.2, *n_b_* = 0.5	3612	0.2573
		*n_t_* = 0.5, *n_b_* = 0.2	3559	0.2575
		*n_t_* = −0.2, *n_b_* = −0.5	2506	0.2628
		*n_t_* = −0.5, *n_b_* = −0.2	2507	0.2608
